# In Silico and In Vitro Evaluation of Bevacizumab Biosimilar MB02 as an Antitumor Agent in Canine Mammary Carcinoma

**DOI:** 10.3390/ani13152507

**Published:** 2023-08-03

**Authors:** Georgina A. Cardama, Paula L. Bucci, Jesús S. Lemos, Candela Llavona, Micaela A. Benavente, Eva Hellmén, María Laura Fara, Eduardo Medrano, Eduardo Spitzer, Ignacio A. Demarco, Patricia Sabella, Juan Garona, Daniel F. Alonso

**Affiliations:** 1 Centro de Oncología Molecular y Traslacional (COMTra), Departamento de Ciencia y Tecnología, Universidad Nacional de Quilmes, Bernal B1876, Argentina; paulabucci1@gmail.com (P.L.B.); jesusselemos@gmail.com (J.S.L.); llavonacandela@gmail.com (C.L.); juan_garona@hotmail.com (J.G.); 2 Plataforma de Servicios Biotecnológicos, Departamento de Ciencia y Tecnología, Universidad Nacional de Quilmes, Bernal B1876, Argentina; 3 Consejo Nacional de Investigaciones Científicas y Técnicas (CONICET), Buenos Aires C1040, Argentina; micaelaabenavente@gmail.com; 4 Centro de Medicina Traslacional (CEMET), Hospital de Alta Complejidad en Red S.A.M.I.C. El Cruce “Nestor Kirchner”, Florencio Varela B5401, Argentina; 5 Laboratorio de Endocrinología, Facultad de Ciencias Veterinarias, Universidad Nacional del Centro de la Provincia de Buenos Aires, Tandil B7000, Argentina; 6 Centro de Investigación Veterinaria de Tandil (CIVETAN), CONICET—CICPBA—Universidad Nacional del Centro de la Provincia de Buenos Aires, Tandil B7000, Argentina; 7 Department of Anatomy, Physiology and Biochemistry, Swedish University of Agricultural Sciences (SLU), 750 07 Uppsala, Sweden; eva.hellmen@slu.se; 8 Laboratorio Elea Phoenix S.A, Los Polvorines B1613, Argentina; maria.fara@elea.com (M.L.F.); eduardo.medrano@elea.com (E.M.); eduardo.spitzer@elea.com (E.S.); 9 mAbxience S.A.U., Buenos Aires B5148, Argentina; ignacio.demarco@mabxience.com; 10 Biogénesis Bagó S.A, Garín B1619, Argentina; patricia.sabella@biogenesisbago.com

**Keywords:** canine mammary carcinoma, bevacizumab, MB02 biosimilar, vascular endothelial growth factor

## Abstract

**Simple Summary:**

Canine mammary carcinomas (CMC) are associated with poor clinical outcomes and high mortality. Vascular endothelial growth factor (VEGF) is a key regulator of angiogenesis and tumor progression in many solid tumors, including mammary carcinomas. The goal of this work was to establish the therapeutic value of MB02 monoclonal antibody biosimilar to bevacizumab that targets VEGF in CMC. For this purpose, first, we were able to predict in silico that bevacizumab was able to recognize and bind canine VEGF. This was confirmed in vitro using an ELISA-based assay. Additionally, canine VEGF-induced microvascular endothelial cell proliferation was inhibited in a concentration-dependent manner by MB02 biosimilar. These results show a high potential for MB02 as a promising therapeutic agent for the management of CMC.

**Abstract:**

Canine mammary carcinomas (CMC) are associated with major aggressive clinical behavior and high mortality. The current standard of care is based on surgical resection, without an established effective treatment scheme, highlighting the urgent need to develop novel effective therapies. Vascular endothelial growth factor (VEGF) is a key regulator of tumor angiogenesis and progression in the majority of solid cancers, including human and canine mammary carcinomas. The first therapy developed to target VEGF was bevacizumab, a recombinant humanized monoclonal antibody, which has already been approved as an anticancer agent in several human cancers. The goal of this work was to establish the therapeutic value of MB02 bevacizumab biosimilar in CMC. First, through different in silico approaches using the MUSCLE multiple-sequence alignment tool and the FoldX protein design algorithm, we were able to predict that canine VEGF is recognized by bevacizumab, after showing an extremely high sequence similarity between canine and human VEGF. Further, by using an ELISA-based in vitro binding assay, we confirmed that MB02 biosimilar was able to recognize canine VEGF. Additionally, canine VEGF-induced microvascular endothelial cell proliferation was inhibited in a concentration-dependent manner by MB02 biosimilar. These encouraging results show a high potential for MB02 as a promising therapeutic agent for the management of CMC.

## 1. Introduction

Mammary carcinoma is the most common cancer in intact female dogs and these tumors are most frequently found in adult animals [1]. Canine mammary carcinomas (CMC) are associated with poor clinical behavior and high mortality. The current standard treatment is surgical resection, and adjuvant therapy is not effective in most cases [2]. Perioperative administration of desmopressin as surgical adjuvant is under clinical scrutiny [3,4] and hormone-based therapy such as tamoxifen is associated with significant adverse effects [5]. Therefore, novel therapeutic strategies are required to improve the life expectancy and quality of life in these dogs. Specific therapies targeting tumor-specific or tumor microenvironmental molecules are being developed for treating CMC [6]. However, at present, there is no established effective treatment scheme for these tumors.

Angiogenesis, the formation of new blood vessels, is a key process in homeostasis and disease. One key angiogenic factor that promotes and orchestrates neovascularization is the vascular endothelial growth factor (VEGF). Uncontrolled angiogenesis can promote numerous diseases, including cancer [7]. In fact, VEGF is overexpressed in various cancers in different species, including human and veterinary mammary tumors [8,9]. In cats bearing mammary tumors, for example, VEGF-A overexpression was shown to be associated with a shorter overall survival. Moreover, cats with aggressive tumor subtypes had elevated serum VEGF-A, VEGFR-1 and VEGFR-2 levels, as well as elevated tumor-infiltrating lymphocytes, highlighting the key role of tumor microenvironment in human and veterinary breast cancer. Indeed, VEGF-A, VEGFR-1 and VEGFR-2 are proposed as potential therapeutic targets and prognostic biomarkers in feline mammary carcinoma [10,11].

In dogs, VEGF receptors appear to be very similar to human VEGF receptor in that they enable a functional growth factor exchange and display identical cell-binding properties [12]. Importantly, VEGF receptor 2 expression and microvascular density are increased in metastatic CMC compared to non-metastatic tumors, indicating a key role of angiogenesis in CMC progression [13]. VEGF has become a main target for angiogenesis inhibition. Blocking the activity of VEGF using specific antibodies to prevent VEGF binding and the activation of its receptors has been one of many approaches used to target angiogenesis. This is the case of bevacizumab (BEV), a recombinant humanized IgG1 monoclonal antibody (mAb) which has already proven to be effective in diverse pathologic settings by inhibiting angiogenesis. BEV has been clinically tested for the treatment of different human cancers, including breast, lung, and colorectal cancer [14,15,16], and it is considered a first- and second-line treatment regimen in combination with chemotherapy for multiple common malignancies [17]. The costs of therapeutic mAbs are often high and veterinary access to them is frequently limited [18]. However, the development of biosimilar mAbs after the expiration of the originators patents makes these therapeutic options more cost-effective and accessible [19]. This is the case of the BEV biosimilar MB02 that has already proven to have pharmacokinetic similarity and bioequivalence to the reference BEV in human clinical trials [20,21].

The aim of the present study was to evaluate whether a BEV biosimilar can become a feasible therapeutic agent for the management of CMC. For this purpose, we performed in silico and in vitro studies to establish the potential activity of MB02 in CMC.

## 2. Materials and Methods

### 2.1. Bioinformatics Analysis

#### 2.1.1. Sequence Alignment

The Generous module was used to analyze the linear amino acid residue sequence of canine (AAD29684.1), feline (XP_023109318.1) and equine (XP_023479773.1) VEGF compared to the sequence of human VEGF (NCBI NP_003367.4). It is important to note that VEGF-A was used in all experiments shown. All available canine VEGF sequences were included in the canine alignment. Generous was used with MUSCLE, a program used to create multiple alignments of amino acid or nucleotide sequences. Default parameters were used showing the best average accuracy in our tests (https://assets.geneious.com/manual/2022.1/static/GeneiousManualsu88.html; accessed on 15 June 2022). Pairwise sequence alignment was performed to match regions in sequences to identify likely structural and functional similarities. Both global alignment methods were used to ensure that each part of the two sequences are aligned (Needleman and Wunsch) and local (Smith and Waterman).

#### 2.1.2. Structural Analysis

Computational studies were carried out to predict the potential interaction of BEV and canine VEGF. The 3D structure of BEV was obtained from Protein Data Bank (PDB) (https://www.rcsb.org/structure/6BFT, file A; accessed on 15 June 2022). Predictions of structural stability were performed by studying enthalpy changes as a result of single point mutations of key residues. FoldX was used to predict energy changes in different VEGF variants (wt vs. mutant). Then, FoldXl was used to calculate the free energy of the wild type (WT) and the mutant (MT) versions (Equation (1)):

ddG(change) = dG(MT) − dG(WT)(1)

FoldX is trained to predict experimental values of ddG(change). It is important to mention that dG(WT) and dG(MT) are meaningless numbers as such. These do not correlate with experimental values. Only ddG(change) does. As a rule of thumb, we use ddG(change) > 0 when the mutation is destabilizing and ddG(change) < 0: when the mutation is stabilizing.

### 2.2. Cell Lines

The canine mammary tumor cell line CMT-U27 was used. This cell line was established from a simple ductal carcinoma at the Swedish University of Agricultural Sciences (SLU) in Sweden [22]. The immortalized human microvascular endothelial cell line HMEC-1 and CMT-U27 cells were maintained in D-MEM (Sigma Aldrich, Buenos Aires, Argentina) or RPMI (Sigma Aldrich), respectively, supplemented with 10% fetal bovine serum (FBS) (Natocor, Córdoba, Argentina), and 10% gentamicin. All cell cultures were maintained at 37 °C and 5% CO_2_ in a humidified incubator. All culture supplies were obtained from Thermo Fisher Scientific, unless otherwise indicated.

### 2.3. Reagents

MB02 (Bevax^®^) Lot: 8113 was provided by Laboratorio Elea Phoenix S.A, Buenos Aires, Argentina. MB02 biosimilar was approved for marketing in the EU in March 2021 by the European Commission and in April 2022 by FDA. MB02 is a biosimilar to BEV, a humanized monoclonal IgG1 antibody designed using recombinant DNA technology and expressed in Chinese hamster ovary (CHO) cells. The following were used: Human VEGF 165 Biotinylated Antibody, RD Systems (code: BAF293), SureBlue TMB1, KPL (code: 52-00-00), Vecstatin ABC Kit, Vector Labs (code: PK-4000), Bevacizumab Secondary Reference Standard RM-BV-01 (mAbxience S.A.U, Buenos Aires, Argentina).

### 2.4. Collection of Tumor-Conditioned Media (TCM) of Canine CMT-U27 Cells

CMT-U27 cells were plated in a complete medium and, after 24 h, the medium was replaced with a serum-free RPMI. After 24 h, the culture supernatant was collected and centrifuged at 5000 rpm for 10 min to remove detached cells and cellular debris. The supernatant was subsequently fractionated and kept at −80 °C for subsequent use.

### 2.5. Canine VEGF Quantitative Assay

Levels of VEGF in CMC cell-conditioned media were quantified using a commercially available, species-specific canine VEGF ELISA Kit (TermoFisher Scientific, Buenos Aires, Argentina), following the manufacturer’s instructions. After incubation, the reaction mixtures were transferred to the ELISA microplates and an analysis for free VEGF was performed. The absorbance was read at 450 nm and corrected by subtracting readings at 540 nm, following the manufacturer’s recommendations.

### 2.6. VEGF Binding Assay (In Vitro Potency Assay)

An in-house competitive ELISA was used to assess the binding of MB02 mAb to canine and feline VEGF. Briefly, human, canine and feline VEGF were incubated with serial dilutions of MB02 or Bevacizumab reference standard. After 90 min of incubation, unbound VEGF was detected using biotinylated anti-VEGF antibody (R&D Biosystems, Buenos Aires, Argentina), followed by the use of ABC Kit (Vector Labs) and substrate incubation. The binding curve reflecting the binding ability of MB02 to species-specific VEGF was obtained, and the EC50 value was calculated. Sample potency was estimated relative to the reference standard (incubated with human VEGF) (potency:100).

### 2.7. TCM-Stimulated Microvascular Endothelial Cell Growth

In order to evaluate the in vitro antiangiogenic effect of MB02 mAb, a 48 h TCM-induced endothelial cell growth assay was performed. First, HMEC-1 cells were seeded into 96-well plates (4000 cells/well) in a complete medium at 37 °C and 5% CO_2_. After 24 h, the depleted culture medium was removed and replaced with a fresh medium supplemented with 1% FBS (final concentration), plus 50 μL of CMT-U27 TCM, in addition to different concentrations of MB02 biosimilar in PBS. In the TCM control group, only the vehicle (PBS) was added. After a 48-h incubation, cell growth was determined using the crystal violet method [23]. The absorbance was determined using excitation and emission wavelengths of 450/595 nm, respectively, in a 96-well Spectra UV/Vis plate reader (ASYS, UVM340). This experiment was repeated at least three independent times.

### 2.8. Statistical Analysis

Statistical analyses were performed using the Graph-Pad Prism 5 software (GraphPad Software, Inc., La Jolla, CA, USA). To compare the differences between two experimental groups, Student’s *t* test was used for a normal distribution of data. In case of there being more than two experimental groups, ANOVA analysis with Tukey’s multiple comparisons post-test was used when a normal distribution of the data was determined. Overall significance was set at *p* < 0.05. The data correspond to at least two or three independent experiments unless stated otherwise. The data were presented as mean ± standard deviation (SD).

PLA3.0 software was used to analyze the binding assay results. A four-parameter logistic (4PL) restricted model was used to calculate EC50 and potency.

## 3. Results

### 3.1. In Silico Analyses Show High Degree of Human, Equine, Canine and Feline VEGF Protein Homology

To analyze the degree of homology for VEGF between different mammalian species, the alignment of the deduced human, canine, feline and equine amino acid sequences was performed using multiple sequence comparison by using the MUSCLE multiple-sequence alignment tool.

**Figure 1:**
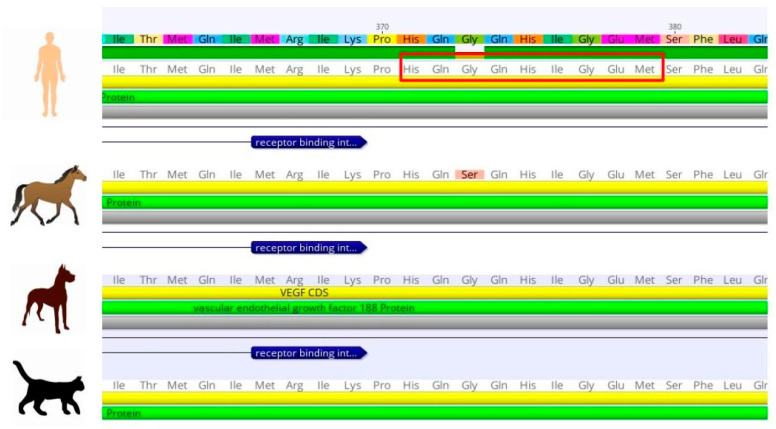
Multiple alignments using amino acid sequences of different isoforms were performed using multiple sequences of VEGFs from different mammalian species. From top to bottom: protein sequences of human NCBI NP_003367.4; equine XP_023479773.1; canine VEGF-A (AAD29684.1); and feline XP_023109318.1.

**Figure 2:**
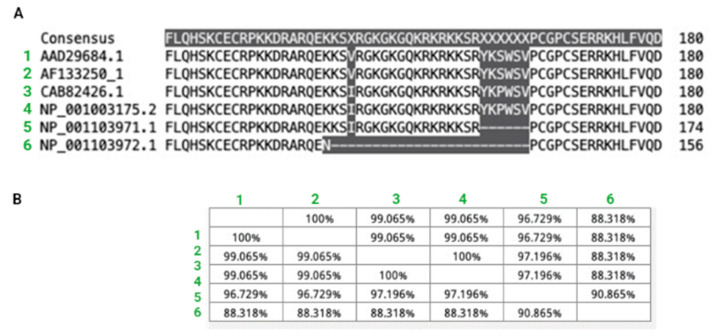
Alignment of the deduced amino acid sequences of all available canine VEGF sequences. (**A**) Comparison of canine VEGF sequences. (**B**) Percentage of similarity between the different aligned VEGF canine sequences. The multiple sequence alignment was performed using Generous Multiple Sequence Alignment Server.

The alignment of the sequences of these species was carried out (Figure 1) and it was possible to observe that they share a high sequence similarity. Globally, this analysis showed a modification of only 4.5% of their amino acid residues and this change is mainly associated with the equine sequences. From this analysis, it could be seen that the canine and human VEGF sequences were identical. To broaden these results, multiple alignments were carried out to analyze all the canine sequences available to date in the gene bank database (Figure 2A). It can be observed that the sequences show similarity (Figure 2B). Only position 161 showed differences in residue identity (highlighted in gray), where serine is replaced by proline and valine by isoleucine. However, based on reports of human VEGF analysis, this region of the sequence is not critical for VEGF recognition and binding by BEV [24].

### 3.2. Computational Analyses Predict BEV Interaction with Canine VEGF

**Figure 3:**
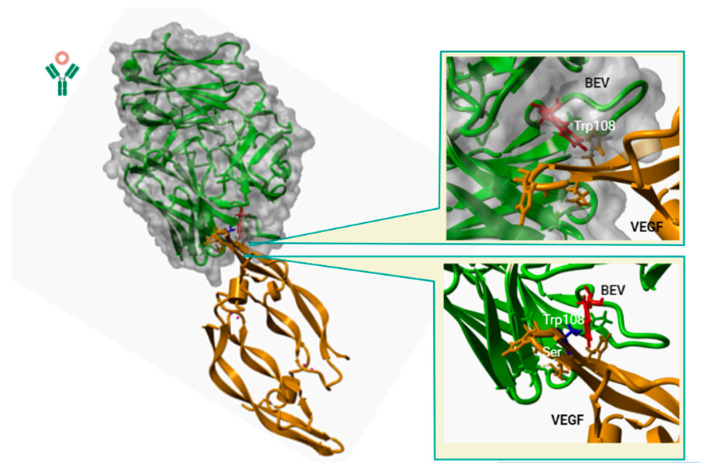
BEV and VEGF interaction. The binding of the antibody and the protein (canine VEGF and BEV) can then be observed in a three-dimensional (3D) image. Interaction between BEV (represented in green color) and the canine VEGF (orange color).

In order to predict the potential binding between BEV and canine VEGF, the interaction between the two molecules was analyzed by using the in silico FoldX protein design tool (Figure 3). Using the molecular modelling of BEV and the canine VEGF interaction, the establishment of close proximity and putative interactions between Trp108 of BEV antibody (represented in red) and Gly113 in canine VEGF was observed, predicting BEV–canine VEGF interaction. These in silico experiments show a strong interaction between BEV and canine VEGF, with a predicted activation energy of −5.66 kcal.mol^−1^.

To further evaluate the importance of Gly at position 88 and the possible effect of amino acid changes in this position, in silico experiments were carried out using single-point mutants, replacing Gly88 for Ser88, a change seen in equine sequence compared to the human sequence. It can be observed that the introduced Ser residue (represented in blue) enables the approach (less than the VsW radius) to theTrp108 of BEV, which could represent the main factor leading to a weaker interaction. This is also seen by the significant activation energy drop for this interaction, reaching predicted values of 3.33 kcal.mol^−1^. The mutation seems to be highly destabilizing for the interaction and this is apparently due to a Van der Waals (VdW) clash.

### 3.3. MB02 BEV Biosimilar Is Able to Bind to Canine VEGF In Vitro

To verify the in silico predictions in regard to the BEV interaction with canine and feline VEGF, we determined the binding capacity of MB02 biosimilar to canine VEGF in vitro using a competitive ELISA assay. As seen in Table 1, different concentrations of canine and feline VEGF were analyzed. MB02 biosimilar was able to recognize and bind to canine and feline VEGF in all tested concentrations. MB02 was able to recognize and bind to canine VEGF. EC50 values around 20 were obtained for 50 ng/mL de VEGF in all species. This is important since EC50 serves as an indication of drug potency. In fact, concentration–response curves were similar for 50 ng/mL of human VEGF and 117.5 ng/mL canine VEGF. MB02 showed to have an equivalent binding capacity to human and feline VEGF, as the potency values fall within the acceptable potency range (between 80 and 120). This confirms the in silico predictions in a highly used and characterized in vitro platform.

### 3.4. MB02 Reduced the Proliferation of Endothelial Cells Exposed to Canine Mammary Carcinoma-Conditioned Media

**Figure 4:**
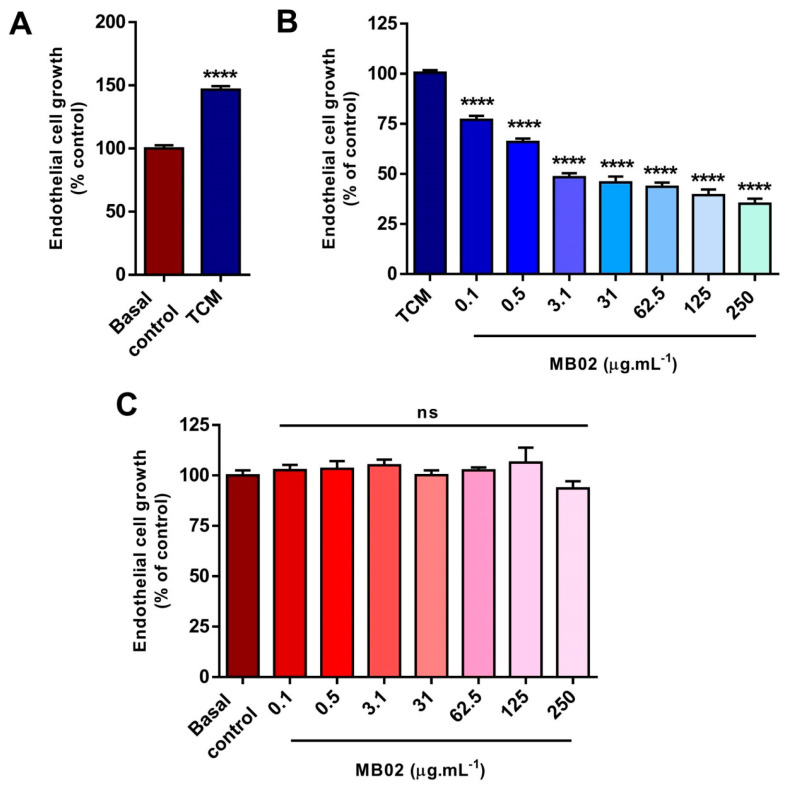
In vitro study of MB02 activity on microvascular endothelial cells stimulated with canine TCM. (**A**) HMEC-1 cells stimulated with canine TCM for 48 h. **** *p* < 0.0001 Student’s *t* test. (**B**,**C**): proliferation of HMEC1 cells stimulated with (**A**) or without (**B**) canine TCM in the presence of different concentrations of MB02 biosimilar. ANOVA cont. Tukey’s (vs. control). **** *p* < 0.0001. ns: non-significant.

To further evaluate the angiostatic effects of MB02 biosimilar in vitro, the cell proliferation of endothelial cells was evaluated after exposure to a tumor cell-conditioned medium. Before assessing MB02 antiangiogenic activity, secreted canine VEGF in the TCM was quantified via ELISA, obtaining an average value of 0.21 ng.mL^−1^ in the evaluated samples. Once canine VEGF presence was confirmed, the impact of TCM from canine mammary carcinoma CMT-U27 cells on the proliferation of HMEC-1 microvascular endothelial cells was assessed. HMEC-1 cells were stimulated with canine TCM for 48 h. As seen in Figure 4A, canine TCM induced a 50% increase in microvascular endothelial cell proliferation, as evaluated via the crystal violet assay. However, we cannot rule out the possibility that other molecules that are present in the CMC-conditioned media have a stimulating activity in these experiments. We are able to affirm that VEGF contributes significantly, but not exclusively. Finally, the in vitro angiostatic effects of MB02 at different clinically relevant concentrations were assessed on HMEC-1 exponentially growing cultures and revealed after a 48-h exposure to the canine conditioned media. As shown in Figure 4B, MB02 was able to significantly inhibit endothelial cell growth induced by VEGF-containing canine TCM in a concentration-dependent manner. It is important to note that MB02 had no effect on endothelial cell proliferation in the absence of VEGF (Figure 4C). These results show that MB02 is able to interfere with canine VEGF-stimulated endothelial cell proliferation, further confirming the in silico predictions and in vitro binding assays.

## 4. Discussion

In veterinary medicine, there has been a long tradition of effectively using human therapeutic tools for veterinary applications. However, it is surprising that only a few biological agents have been approved for veterinary medicine, including Frunevetmab and Bendinvetmab, anti-nerve growth factor antibodies approved for pain control in cats and dogs with osteoarthritis, respectively, and Lokivetab, an anti-interleukin-31 antibody for the treatment of pruritus and atopic dermatitis in dogs [25]. The importance of biological agents, in particular monoclonal antibodies (mAbs), as therapeutic agents is increasing and, currently, there is an average of four new molecules reaching the human therapeutics’ market every year for the treatment of several diseases, including cancer [18]. Thus, there is a clear opportunity to use mAbs as therapeutic agents for veterinary diseases, particularly in cats and dogs.

There are still many challenges to overcome in order to achieve the commercial viability and wide accessibility of human-approved mAbs for veterinary use. First, mAbs are usually species-specific since they are designed to recognize specific human epitopes and usually do not cross-react with the canine or feline epitopes. Second, the high costs of mAb therapies make their veterinary application much more challenging and limited. Therefore, target validation in cats and dogs, and the determination of cross-reactivity between species are imperative for the development of mAb-based therapies in veterinary medicine. Additionally, there is an interesting opportunity for the use of biosimilar antibodies in veterinary medicine due to their significantly lower prices, making mAb therapies more accessible.

Angiogenesis is a key event during tumor progression, with VEGF being one of its tractable inducers. One of the main activities of VEGF is the ability to promote the growth of vascular endothelial cells derived from arteries, veins, and lymphatics. Therefore, blocking the activity of VEGF to prevent its binding and signaling through its receptors has been a valuable strategy to reduce tumor growth. BEV, a VEGF-targeting monoclonal antibody, was the first approved anti-angiogenesis inhibitor [26]. It was approved for a broad range of human solid tumor indications and has also demonstrated efficacy in the management of retinal disease [27,28]. The price of complex biological agents such as BEV is often high mainly due to the enormous cost (and low success rate) of bringing such novel therapeutic agents to the market [29]. However, the recent expiration of patents for some of these oncologic mAb has instigated the global development of biosimilars to compete with their reference medicinal products, increasing the access to this valuable therapy [30] and presenting a critical opportunity to test its clinical efficacy in non-human tumors.

It is important to point out that VEGF is a pleiotropic growth factor that modulates neovascularization not only in the tumor, but also in the embryo and the adult. In fact, the perturbation of VEGF signaling may impact organ homeostasis in several ways. Endothelial fenestration, vascular permeability, vessel turnover and organ perfusion are some of the key activities regulated by VEGF in adult organs. The loss of VEGF function could potentially lead to tissue injury [31]. In fact, the side effects of anti-VEGF therapies for human cancers are downstream effects of the suppression of VEGF signaling in endothelial cells of normal organs, causing bleeding, hypertension and asymptomatic proteinuria [32]. Importantly, anti-VEGF therapy is well-tolerated and many of these effects are reversible, thus being a promising strategy for tumor treatment.

The main goal of this work was to validate a BEV biosimilar, MB02, already approved for human use by stringent regulatory authorities as a possible therapeutic agent in canine mammary cancer. One key issue of the process of the development of biosimilars is the appearance of heterogeneous charge variants during the manufacturing process. An extended characterization of MB02 individual charge variants of MB02 and its comparison with the reference mAb have recently been reported. MB02 has demonstrated analytical and functional similarity to reference BEV [33]. Additionally, PK similarity has been further confirmed in three bioequivalence studies comparing the pharmacokinetic profiles of MB02 in healthy volunteers [20,34,35]. Additionally, a phase III study was conducted comparing both antibodies in advanced non-small cell lung cancer, showing comparable safety and immunogenicity profiles [21].

First, to assess and confirm BEV cross-reactivity in canines, an updated analysis of canine and human VEGF sequences was carried out. We first confirmed that human and canine VEGF sequences are almost identical, as described previously by Scheidegger et al. [12]. In fact, the key 19-residue sequence involved in BEV recognition [24] shows 100% homology in all available canine sequences. This is not the case for the horse VEGF sequence, which showed a change in the Gly113 residue for a Serine113. It should be mentioned that in the structural alignment, Gly113, corresponding to the alignment shown above, corresponds to Gly88. The change in numbering refers to a different nomenclature used in the structural alignment. However, it is the same amino acid. In previous reports, it has been shown that Gly113 is a key residue responsible for the species-specific VEGF-BEV binding, having a profound impact on VEGF recognition [24,36]. In fact, it has already been reported that BEV is unable to recognize and bind to horse VEGF in vitro [37]. Further, this residue change has already been shown to be the main determinant for the lack of recognition of BEV for murine VEGF [38]. Given the fact that canine VEGF is almost identical to the human variant, BEV strong recognition of canine VEGF was expected and further confirmed using in silico predictions.

Previous preclinical studies show a potential use of BEV in different canine cancer settings. For instance, BEV showed antitumor activity in canine osteosarcoma [39] and canine hemangiopericytoma [40] murine models. These results reinforce the idea of species cross-reactivity of BEV and its potential use in canine cancer.

The fact that MB02 biosimilar already demonstrated analytical and functional similarity to reference BEV, together with the prediction of BEV recognition of canine VEGF, provided strong support for the idea of MB02 use in dogs. In this work, after confirming its binding capacity to canine VEGF, we showed that MB02 elicits a potent angiostatic activity in vitro, reducing the growth of microvascular endothelial cells in the presence of CMC-conditioned media. It is worth noting that this VEGF-containing TCM had a significant growth-stimulating effect on endothelial cell cultures, and that the VEGF concentration determined in these conditioned media was in the expected concentration range for the CMT-U27 cell line [41] and other canine tumor cell lines [42]. Interestingly, we observed that incubation with MB02, especially at the micromolar concentration range, was capable of impairing HMEC-1 growth below basal levels (without addition of CMT-U27-conditioned media). Despite the fact that VEGF-containing TCM had a net stimulatory effect on endothelial cell proliferation, it must be borne in mind that such conditioned media contains a complex mixture of both pro- and antiangiogenic factors secreted by canine tumor cells [43]. In this setting, these endogenous tumor cell-derived inhibitors of angiogenesis could, in part, explain this increased angiostatic effect after VEGF recognition and sequestration by MB02. Additionally, it is also important to mention that MB02 showed a complete lack of activity in the absence of CMC-conditioned media. The use of additional canine carcinoma cell lines to collect conditioned media from different origins would be important to establish the reproducibility of these results since different cell-associated secretomes could impact the degree of cell proliferation stimulation.

It is important to highlight the limitations of this study. This work provides a first approach to establish the therapeutic value of MB02 in the management of CMC. More preclinical studies are necessary to determine efficacy and toxicology of MB02 in CMC. Importantly, there is a need to characterize the effects of using humanized antibodies in pets. Hurdles have been described mainly due to the intrinsic mechanism of action of mAb, where side effects can arise as a consequence of a host reaction against the antibody. Additionally, not all monoclonal antibodies cross-react with Fc receptors from other species, limiting the effectivity of this therapeutic approach due to a rapid clearance of the circulating antibody. To overcome some of these limitations, speciation is proposed, a lengthy and labor-intensive laboratory process that is not always feasible [18].

However, it has been described that human IgG1 is able to bind to canine receptor FcγRI; therefore, they are potentially useful as therapeutics in dogs [44], with MB02 being a recombinant humanized IgG1 mAb. Additionally, there are some examples of licensed antibodies that are used in humans and are not humanized (anti-CD20 antibodies), leaving an opportunity for using non-speciated mAbs in animals. This still needs to be addressed in further studies.

## 5. Conclusions

These in silico and in vitro results pave the way to further explore the potential of MB02 for the management of CMC, highlighting a unique opportunity for mAbs biosimilars as novel therapeutic tools in veterinary medicine. Clinical studies in dogs to investigate the efficacy and tolerance of MB02 are warranted.

## Figures and Tables

**Table 1:** Biosimilar to canine and feline VEGF analyzed via competitive ELISA.

Species	VEGF Concentration (ng/mL)	EC50 * (ng/mL)	Potency **
Human	50	20.61	100
Canine	50	19.73	179.61
	65	18.72	189.66
	80	21.04	145.84
	107.5	25.68	108.2
	110	22.6	111.88
	112.5	26.95	93.3
	117.5	25.62	97.16
	120	24.13	95.88
	130	25.52	88.51
Feline	50	18.04	117.1
	55	23.78	96.17
	60	25.94	83.82
	65	24.07	82.8
	80	30.55	60.13

* EC50: half maximal effective concentration; ** relative to reference standard (100%).

## Data Availability

The data presented in this study are available upon request from the corresponding authors.
